# Nestin Expression Affects Resistance to Chemotherapy and Clinical Outcome in Small Cell Lung Cancer

**DOI:** 10.3389/fonc.2020.01367

**Published:** 2020-08-06

**Authors:** Kazuki Sone, Ken Maeno, Ayako Masaki, Eiji Kunii, Osamu Takakuwa, Yusuke Kagawa, Akira Takeuchi, Satoshi Fukuda, Takehiro Uemura, Kensuke Fukumitsu, Yoshihiro Kanemitsu, Hirotsugu Ohkubo, Masaya Takemura, Yutaka Ito, Tetsuya Oguri, Hiroshi Inagaki, Akio Niimi

**Affiliations:** ^1^Department of Respiratory Medicine, Allergy and Clinical Immunology, Nagoya City University Graduate School of Medical Sciences, Nagoya, Japan; ^2^Department of Pathology and Molecular Diagnosis, Nagoya City University Graduate School of Medical Sciences, Nagoya, Japan; ^3^Department of Respiratory Medicine, Nagoya City West Medical Center, Nagoya, Japan; ^4^Department of Education and Research Center for Community Medicine, Nagoya City University Graduate School of Medical Sciences, Nagoya, Japan

**Keywords:** nestin, small cell lung cancer, chemotherapy, resistance, immunohistochemistry

## Abstract

**Objectives:** Small cell lung cancer (SCLC) is an aggressive and highly metastatic lung cancer subtype. Nestin is a member of the intermediate filament family and serves as a potential proliferative and multipotency marker in neural progenitor and stem cells. Aberrant expression of nestin is linked to poor prognosis in different cancers, including non-small cell lung cancer. However, the association between nestin expression and clinicopathological feature or prognosis has remained unclear for SCLC. This study examined whether nestin expression was associated with malignant features and clinical outcomes in SCLC.

**Materials and Methods:** Using previously established *Nestin* knock-down cells and a newly established *Nestin*-overexpressing cell line, we examined the relationship between nestin expression and cell proliferation *in vitro* and *in vivo* and chemosensitivity. We also analyzed nestin expression in three drug-resistant lung cancer cell lines. Furthermore, we examined samples from 84 SCLC patients (16 patients with surgical resection, and 68 patients with biopsy), and immunohistochemically analyzed nestin expression.

**Results:** Nestin expression correlated positively with cell proliferation, but negatively with chemosensitivity. Nestin expression in drug-resistant cell lines was upregulated compared to their parental cells. Among the 84 SCLC patients, 24 patients (28.6%) showed nestin-positive tumor. Nestin-positive ratio tended to be higher in operated patients than in biopsied patients. Nestin-positive and -negative patients showed no significant differences in response rate (RR) or progression-free survival (PFS) following first-line chemotherapy. However, positive expression of nestin was associated with shorter PFS following second-line chemotherapy (median PFS: nestin-positive, 81 days vs. nestin-negative, 117 days; *P* = 0.029).

**Conclusions:** Nestin expression may be associated with malignant phenotype and worse outcome in SCLC patients.

## Introduction

Small cell lung cancer (SCLC) is an aggressive, highly metastatic lung cancer subtype, and represents 13% of all newly diagnosed cases of lung cancer (1). The majority of SCLC patients already show metastasis by the time of diagnosis, so chemotherapy accounts for a large proportion of the initial treatment options for SCLC. Although SCLC is highly sensitive to chemotherapy, development of drug resistance is frequent during the disease course and leads to a high relapse rate. The use of small-molecule tyrosine kinase inhibitors and immunotherapy have led to large survival benefits in patients with advanced non-small lung cancer (NSCLC) over the past 2 decades (2). Recently, PD-L1 blockade adding on chemotherapy prolonged survival of SCLC patients (3). However, the additional survival benefit wasn't so long. Therefore, survival times in SCLC patients have not significantly improved since the 1980s (1). New treatment strategies for SCLC are thus needed to improve survival.

Nestin is a member of the intermediate filament family and serves as a potential proliferative and multipotency marker in neural progenitor and stem cells (4). We have previously reported nestin as associated with neuroendocrine features and participating in malignant phenotypes, including cell growth. In addition, nestin was detected in SCLC tumor cells from clinical specimens (5). Nestin expression in tumor cells reportedly led to chemoresistance in a hepatocellular carcinoma cell line (6) and radioresistance in a nasopharyngeal carcinoma cell line (7). Moreover, nestin expression was also related to poor prognosis in both patients with resected NSCLC treated with adjuvant chemotherapy (8) and patients with resected lung large cell neuroendocrine carcinoma (9). We therefore assumed that nestin expression in SCLC also leads to an aggressive phenotype and chemoresistance, and would thus be associated with the efficacy of chemotherapy in the clinical setting. The present study therefore examined the significance of nestin expression in SCLC using SCLC cell lines and clinical samples.

## Materials and Methods

### Cell Lines and Chemicals

We used three human lung small-cell carcinoma lines (PC6, DMS53, and SKLC17), and two human adenocarcinoma cell lines (PC9 and PC14). Their previously described cisplatin-resistant sublines (PC6/CDDP, PC9/CDDP and PC14/CDDP), SN-38-resistant sublines (PC6/SN38), etoposide-resistant sublines (PC6/ETP) and nestin knock-down and control cell lines (DMS53 shNES-1, shNES-2 and DMS53 ctrl) were also used (5, 10–12). Cells were cultured in RPMI-1640 medium supplemented with 10% heat-inactivated fetal bovine serum and 1% (v/w) penicillin/streptomycin in a humidified chamber (37°C, 5% CO_2_). Drug-resistant sublines were also cultured in this medium with the addition of each drug, and nestin knock-down and control cell lines were also cultured with the addition of blasticidin (6 μg/ml).

### Overexpression of Nestin in SKLC17 Cells

The plasmid pCMV6-Entry/nestin was obtained by subcloning the full-length human nestin cDNA into the pCMV6-Entry mammalian expression vector (OriGene, Rockville, MD). The *Nestin* gene was under the control of the CMV promoter, allowing constitutive expression of nestin. SKLC17 cells (2 × 10^5^ cells/plate) were seeded in a 6-well plate and cultured for 24 h before transfection. Cells were transfected with pCMV6-Entry/nestin or control plasmids (20 μg) using the X-tremeGENE Transfection Reagent (Roche Applied Science, Indianapolis, IN) in serum-free Opti-MEM (Invitrogen, Carlsbad, CA) in accordance with the instructions from the manufacturer. After selection with G418 (500 μg/ml), proteins were extracted from each cell, and nestin expression was analyzed by Western blotting. After confirming increased expression of nestin, transfected cells were cloned in the presence of G418. Subsequently, two cell lines were selected from the transfected cells as nestin-overexpressing cell lines (SKLC17 NES-1 and NES-2). In the same way, cells transfected with the negative control plasmid were cloned, and one cell line that showed similar nestin expression to the parental cells was selected as the control cell line (SKLC17 ctrl). For the *in vitro* cell proliferation assay, 1 × 10^4^ cells were cultured in 6-cm dishes. Cells were trypsinized and counted after 48, 72, and 96 h.

### Protein Extraction and Western Blotting

Equal amounts of total cell lysates were solubilized in sample buffer (50 mM Tris-HCL (pH 6.8), 2% SDS, 1 mM EDTA, and 10% glycerol) with Complete Mini Protease Inhibitor Cocktail Tablets (Roche Diagnostics, Mannheim, Germany) and PhosSTOP Phosphatase Inhibitor Cocktail Tablets (Roche Diagnostics). Subsequently, these lysates were electrophoresed on 4–20% Ready Gel Tris-HCl Precast Gels (Bio-Rad Laboratories, Hercules, CA), then transferred onto Immobilon-P filters (Millipore, Billerica, MA). These filters were first incubated with primary antibodies against nestin (155 kDa) and α-tubulin (50 kDa) at room temperature overnight, then with horseradish peroxidase (HRP)-conjugated secondary antibodies for 1 h. The following antibodies were used: anti-nestin (clone 10C2) (Millipore), anti-α-tubulin (Sigma Aldrich Biotechnology, St. Louis, MO) and HRP-conjugated secondary antibody (Cell Signaling Technology, Danvers, MA). The specific antibody-antigen complexes were detected using an ECL detection system (GE Healthcare Bioscience, Fairfield, CT). This study used α-tubulin as a loading control.

### Mouse Xenograft Model

Male severe combined immunodeficiency (SCID) mice aged 5 weeks were inoculated subcutaneously with 5 × 10^5^ nestin knock-down or control cells in the flank. Mice were maintained at a 12 h light/dark cycle, and allowed to feed *ad libitum* on laboratory chow and water. Tumor size was measured using calipers. All procedures carried out in mice were approved by the Institutional Animal Care and Use Committee at Nagoya City University of Medical Science.

### Drug Sensitivity Assays

Cells were cultured at 5,000 cells/well in 96-well tissue culture plates. To assess cell viability, stepwise 10-fold dilutions of anti-cancer drug were added 2 h after plating, and cultures were incubated at 37°C for 72 h. At the end of the culture period, 20 μl of MTS [3-(4,5-dimethylthiazol-2-yl)-5-(3-carboxymethoxyphenyl)-2-(4-sulfophenyl)-2H-tetrazolium, inner salt] solution (CellTiter 96^®^ AQueous One Solution Cell Proliferation Assay, Promega, Madison, WI) was added, and cells were incubated for a further 3 h. Finally, absorbance was measured at 490 nm using an ELISA plate reader. Mean values were calculated from three independent experiments carried out in quadruplicate. Chemosensitivity is expressed as the drug concentration resulting in 50% growth inhibition (half-maximal inhibitory concentration, IC50), determined using Graph Pad Prism version 4 software (GraphPad Software, San Diego, CA).

### Study Population

This study also included 84 patients with SCLC, who had been diagnosed and treated at Nagoya City University Hospital between January 2010 and September 2016. All patients provided written informed consent in accordance with the Declaration of Helsinki, and the present study was approved by the Institutional Ethics Committee of Nagoya City University Graduate School of Medical Sciences (approval no. 00000 982-2). All routine medical data were anonymized. In our study, the definition of limited disease (LD) was a disease confined to one hemithorax, mediastinum or bilateral supraclavicular fossae. Extensive disease (ED) was defined as other than LD. Other eligibility criteria included age (≥18 years old), normal liver function, and Eastern Cooperative Oncology Group (ECOG) performance status ≤ 2. Every patient included in this study had received at least one chemotherapy regimen.

### Histologic and Immunostaining Analysis

Hematoxylin and eosin (HE) staining and immunostaining analyses were performed on formalin-fixed, paraffin-embedded sections of the affected tissues of 84 SCLC patients (16 patients with surgical resection, and 68 patients with biopsy). Nestin immunostaining was performed using mouse anti-human nestin mAb (clone 10C2) (Millipore) and a BOND-MAX IHC instrument system (LEICA Biosystems, Nussloch, Germany). Peritumor vascular endothelial cells were used as an internal positive control. Positivity was scored as positive if ≥1% of SCLC cells were stained.

### Statistical Analysis

Differences in cell growth between samples were evaluated using the Mann-Whitney U test. Differences in the clinical characteristics of patients according to nestin expression were evaluated by Fisher's exact test. Efficacy was assessed by measurable disease based on Response Evaluation Criteria in Solid Tumors (RECIST) version 1.0. Differences in response rate (RR) and disease control rate (DCR) were evaluated using Fisher's exact test. Survival curves for progression-free survival (PFS) based on nestin expression were calculated using the Kaplan-Meier method and were compared using the log-rank test. All statistical analyses of clinical data were performed with EZR (Saitama Medical Center, Jichi Medical University, Saitama, Japan), a graphical user interface for R (The R Foundation for Statistical Computing, Vienna, Austria). More precisely, EZR is a modified version of R Commander designed to add statistical functions frequently used in biostatistics (13).

## Results

### Expression of Nestin Is Related to Growth of SCLC Cells *in vitro* and *in vivo*

**Figure 1:**
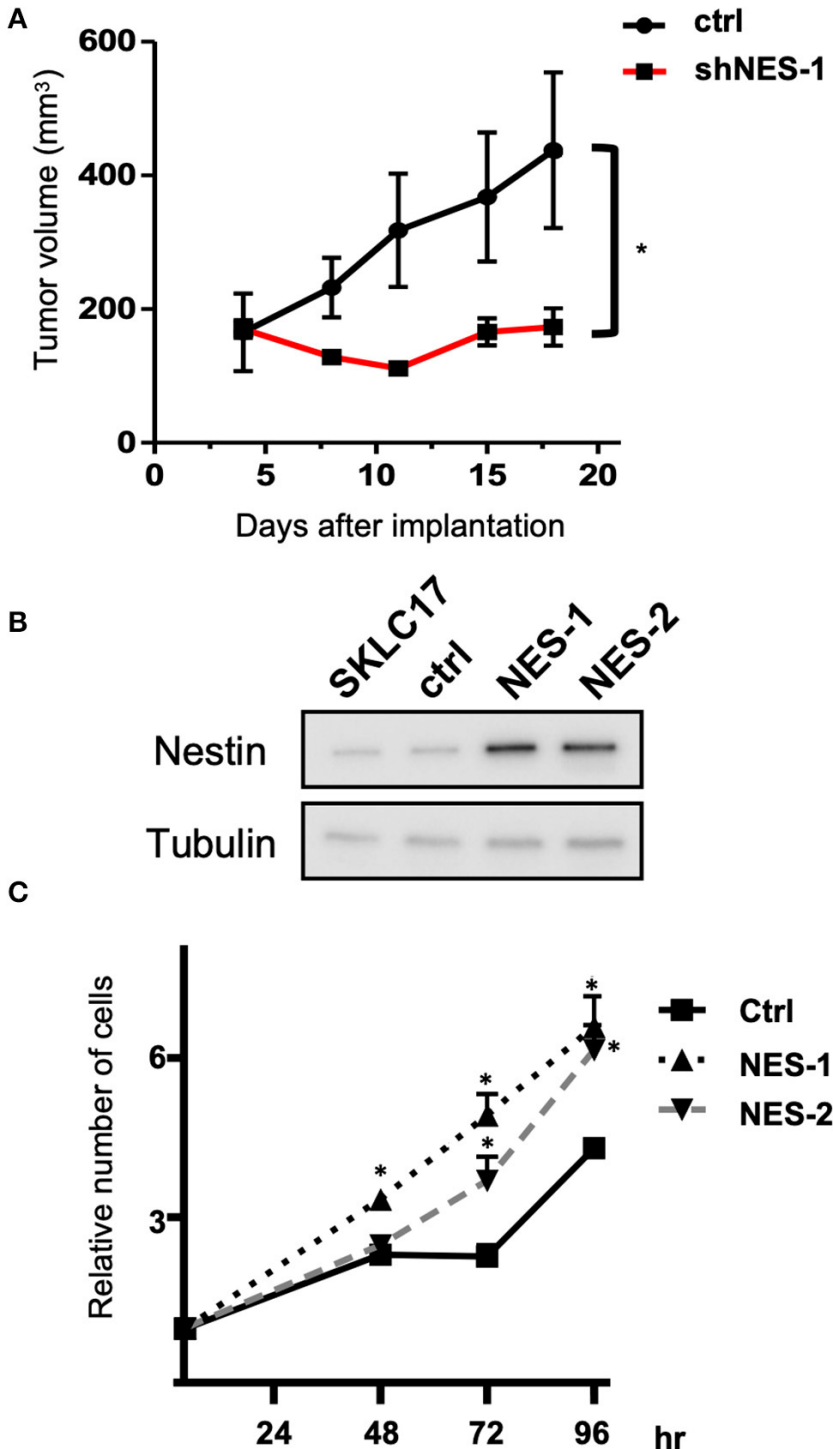
**(A)** Nestin knock-down DMS53 cells (shNES-1) or control cells (ctrl) were implanted subcutaneously in the flank of SCID mice. Tumor volume was significantly smaller for nestin knock-down DMS53 cells than for control cells from 6 days or more after treatment. ^*^P < 0.01. **(B)** Western blotting shows that nestin protein levels are significantly increased in SKLC17 cells treated with nestin overexpression vector (NES-1 and NES-2) when compared with cells transfected with control plasmid (Ctrl). Alpha-tubulin is assayed as a loading control. **(C)** There are significantly more nestin-overexpressing SKLC17 cells than control cells after 48, 72, and 96 h of culture. ^*^P < 0.001.

To confirm the reduced cell growth with knock-down of nestin *in vitro* seen in our previous study (5), DMS53 shNES-1 and ctrl cells were implanted in SCID mice. Tumor growth of DMS53 shNES-1 was significantly inhibited compared with ctrl cell (Figure 1A). Next, we established nestin-overexpressing SKLC17 cells (SKLC17 NES-1 and SKLC17 NES-2) and control cells (SKLC17 ctrl) using plasmid vectors (Figure 1B). In the cell count experiments, cultures showed significantly more NES- 1 and NES-2 cells compared with Ctrl cells (Figure 1C). These findings indicate that nestin expression confers increased proliferation capability on SCLC cells.

### Nestin Expression Affects Anticancer Drug Sensitivity

**Figure 2:**
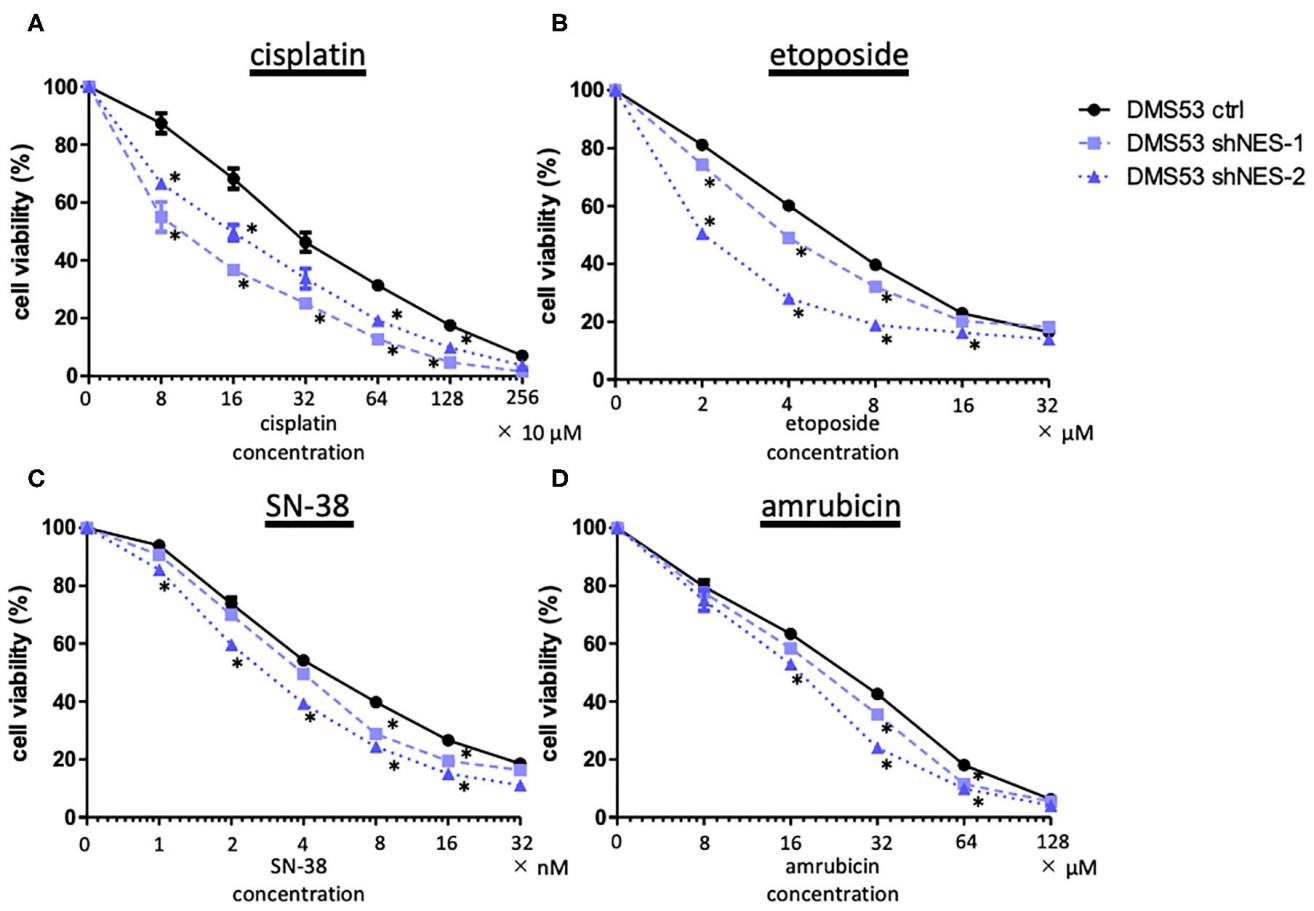
MTS assay demonstrates decreased cell viability in nestin knock-down cell lines (DMS53 shNES-1, shNES-2) compared to control cells (DMS 53 ctrl). **(A)** Cisplatin; **(B)** etoposide; **(C)** SN-38 (irinotecan metabolite); **(D)** amrubicin. ^*^p < 0.05.

Next, we examined the relationship between expression of nestin and the *in vitro* cytotoxicity of anticancer drugs (cisplatin, etoposide, SN-38 and amrubicin, as drugs commonly administered to SCLC patients) using nestin knock-down cell lines and their parent cells. The cytotoxicity of all four drugs tested was higher for nestin knock-down cells than for control cells (Figure 2). In the same manner, cytotoxicity was lower for nestin-overexpressing cells than for control cells (data not shown).

### Nestin Expression in Anticancer Drug-Resistant Cells

**Figure 3:**
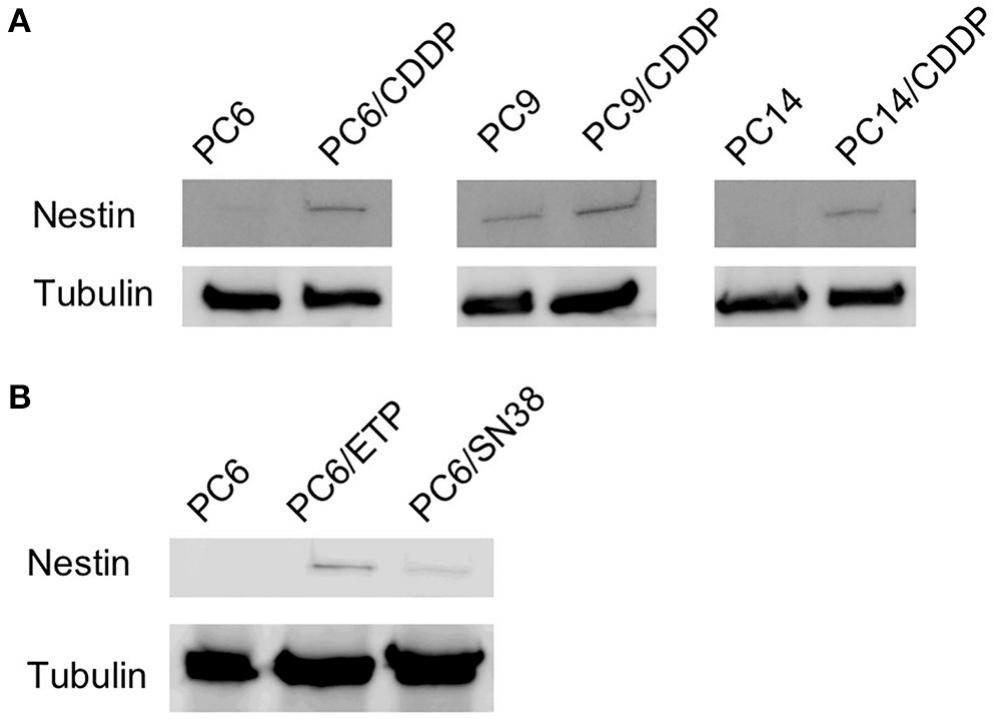
Comparison of nestin protein levels between three cisplatin-resistant cell lines (PC6/CDDP, PC14/CDDP and PC9/CDDP) and their parental cell lines **(A)** and between etoposide-resistant cell lines (PC6/ETP) or SN38-resistant cell lines (PC6/SN-38) and their parental cell lines **(B)** using western blotting. Alpha-tubulin is assayed as a loading control.

Nestin protein expression was relatively higher in three cisplatin-resistant cell lines (PC6/CDDP, PC9/CDDP, and PC14/CDDP) than in the parent cell lines. Moreover, etoposide-resistant cells (PC6/ETP) and SN-38-resistant cells (PC6/SN-38) also displayed higher nestin expressions than their respective parent cells (Figure 3). These data suggest SCLC cells without nestin expression or with low level nestin expression can get nestin expression by continuous exposure of cytotoxic anticancer agents.

### Clinical Characteristics of SCLC Patients According to Immunohistochemical Expression of Nestin

**Figure 4:**
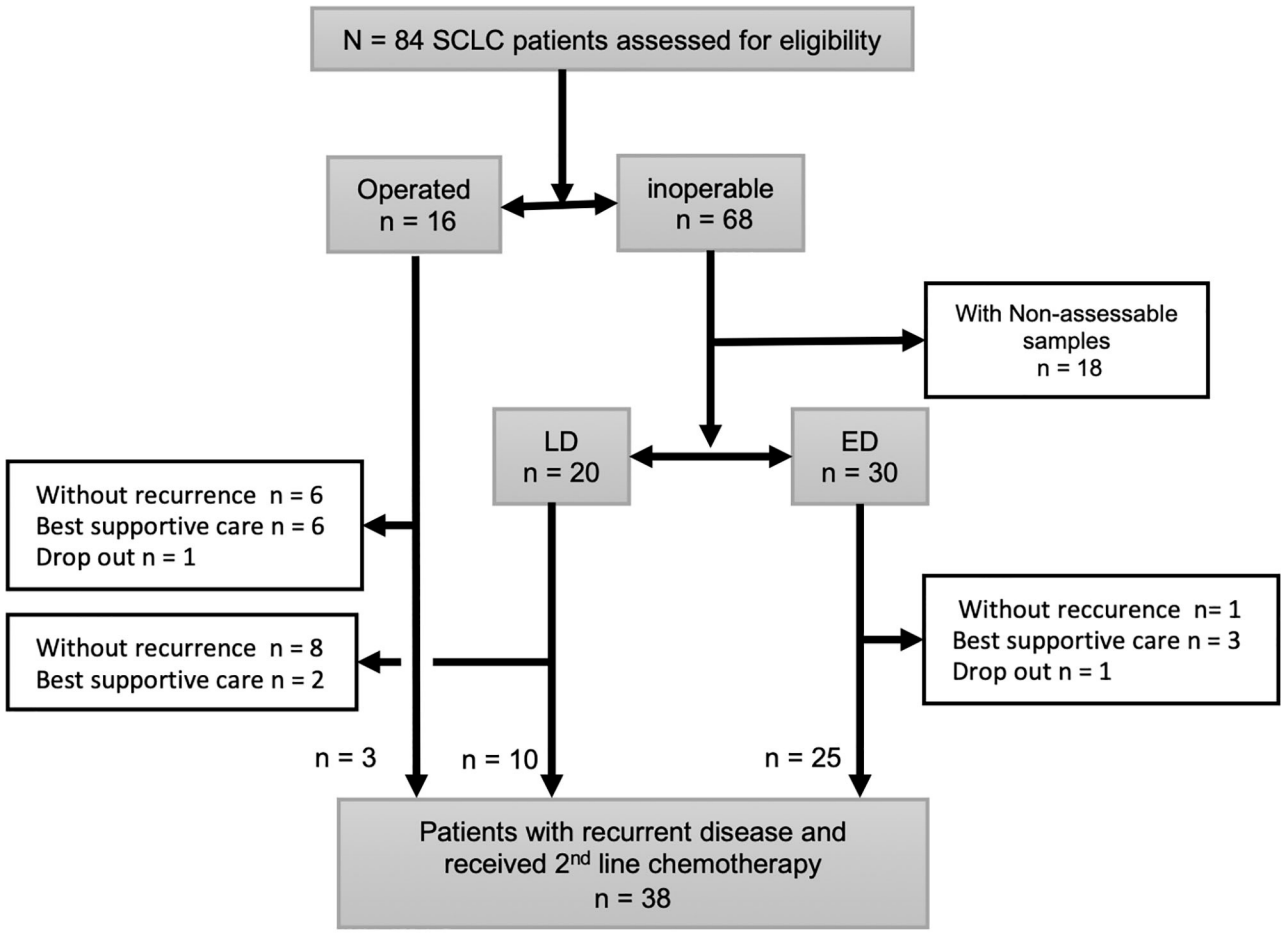
Schema of patients included in this study. All small cell lung cancer (SCLC) patients diagnosed surgically (*n* = 16) or with biopsy (*n* = 68). Eighteen inoperable patients were excluded because immunostainings of their specimens were not evaluable. Total 38 patients had disease recurrence after 1st line therapy and received 2nd line chemotherapy.

**Figure 5:**
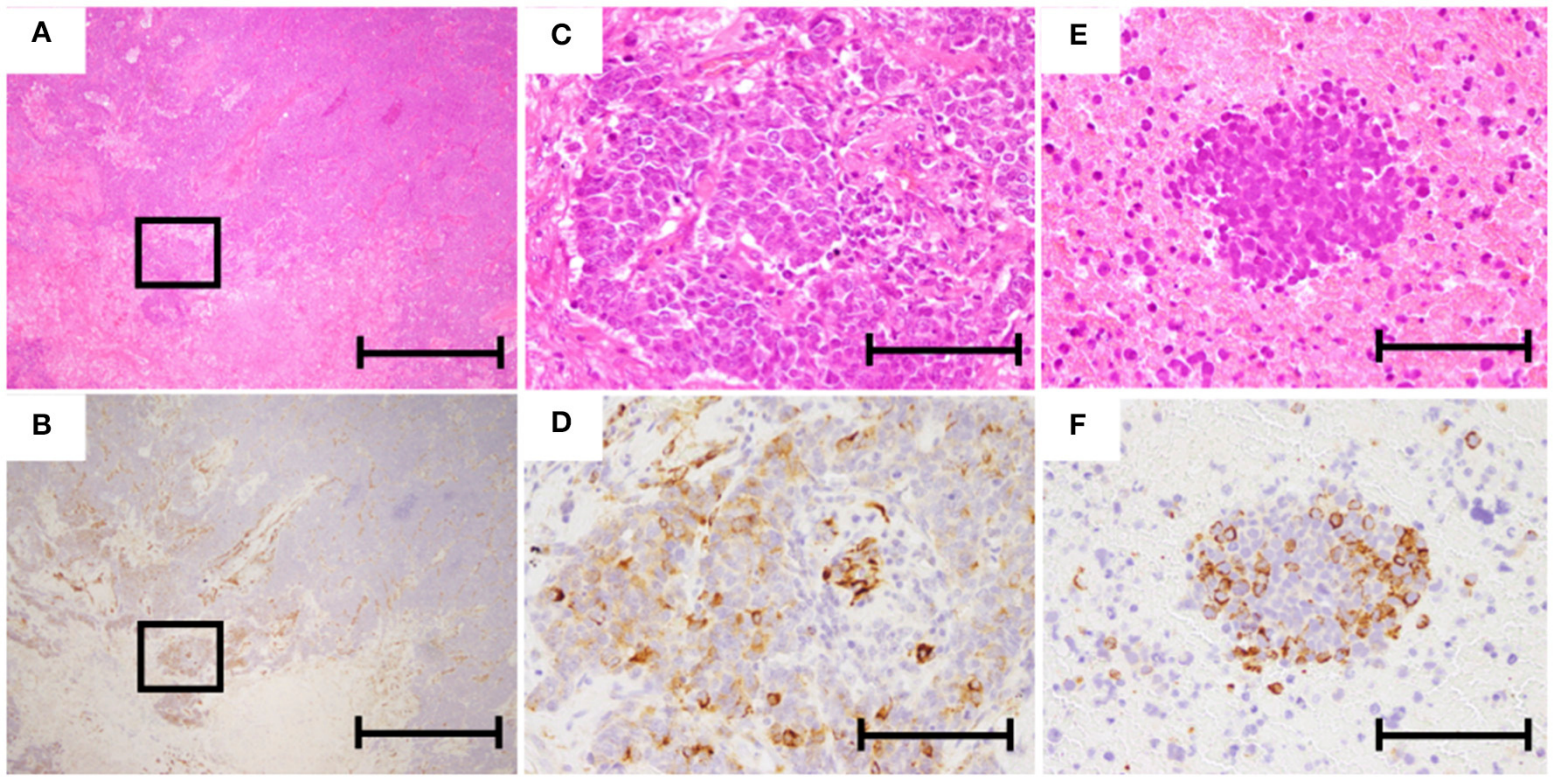
Representative images of hematoxylin and eosin (HE) and immunohistochemical staining of nestin. **(A,C)** HE staining of operated specimens (**A**, ×40; **C**, ×200). **(B,D)** Nestin staining of operated specimen (**B**, ×40; **D**, ×200). **(E)** HE staining of biopsy specimen (×200). **(F)** Nestin staining of biopsy specimen (×200). The scale bars in **(A,B)** represent 1 mm and those in **(C–F)** represent 200 μm.

During the study period, 84 SCLC patients eligible for this retrospective study were treated in our hospital. Of these, 16 patients underwent surgery and received adjuvant chemotherapy. Sixty-eight patients were inoperable and diagnosed from biopsy. Eighteen patients were excluded from the analysis because immunostainings of the samples were not evaluable. Among the 50 inoperable patients, 20 patients showed LD and 30 patients had ED. The 50 inoperable patients received chemotherapy alone or chemoradiotherapy. A total of 38 patients experienced disease recurrence and received second-line chemotherapy. Patient recruitment is summarized in Figure 4. Typical examples of nestin immunostaining are shown in Figure 5. The nestin-positive ratio tended to be higher for surgical specimens (56%) than for biopsy specimens (30%, *P* = 0.076). The characteristics of patients according to nestin expression are summarized in Table 1. Among the 16 operated patients, median age was lower for nestin-positive tumors (70 years) than for nestin-negative tumors (76 years; *P* = 0.023). No significant differences in sex, smoking status, stage or serum Pro gastrin releasing peptide (ProGRP) concentration according to nestin expression were identified in operated patients. Inoperable patients showed no significant difference in age, sex, smoking status, stage or serum ProGRP levels according to nestin expression (Table 1). In patients with recurrent disease, 14 patients showed positive nestin expression, and 24 patients had negative nestin expression. No significant difference in the form of disease progression (sensitive relapse or refractory relapse) were seen according to nestin expression (nestin positive vs. negative; sensitive/refractory 4/10 vs. 9/15, respectively; *P* = 0.73). Treatment regimens according to nestin expression are summarized in Table S1. All ED-SCLC patients were treated with platinum-based chemotherapy as first-line therapy. In patients with recurrent disease, 10 patients (71.4%) with positive nestin expression were treated with amrubicin as second-line therapy, and 18 patients (75%) with negative nestin expression were treated with the same drug (*P* = 0.43).

**Table 1:** Characteristics of the 16 operated SCLC patients and 50 inoperated SCLC patients according to nestin expression.

**Factor**		**Operated patients (*****n*** **=** **16)**			**Inoperaed patients (*****n*** **=** **50)**		
		**Nestin +**	**Nestin –**	***P***	**Nestin +**	**Nestin –**	***P***
Age (years)	Median	70	76	0.023	67	70	0.443
	Range	58–75	65–83		47–85	44–83	
Sex (*n*)	Male	8	6	1	11	31	0.22
	Female	1	1		4	4	
Smoking status (pack.years)	Median	80	45	0.222	50	50	0.865
	Range	25–104	23–104		10–110	10–110	
Stage (*n*)	LD	9	7	1	6	14	1
	ED	0	0		9	21	
Serum ProGRP (pg/ml)	Median	25.5	39.4	0.211	967	710	0.441
	Range	9.3–76.4	14.0–120		39.6–11,200	29.8–18,900	

*SCLC, small cell lung cancer; LD, limited disease; ED, extensive disease*.

### Relationship Between Nestin Expression and Effects of Chemotherapy

**Figure 6:**
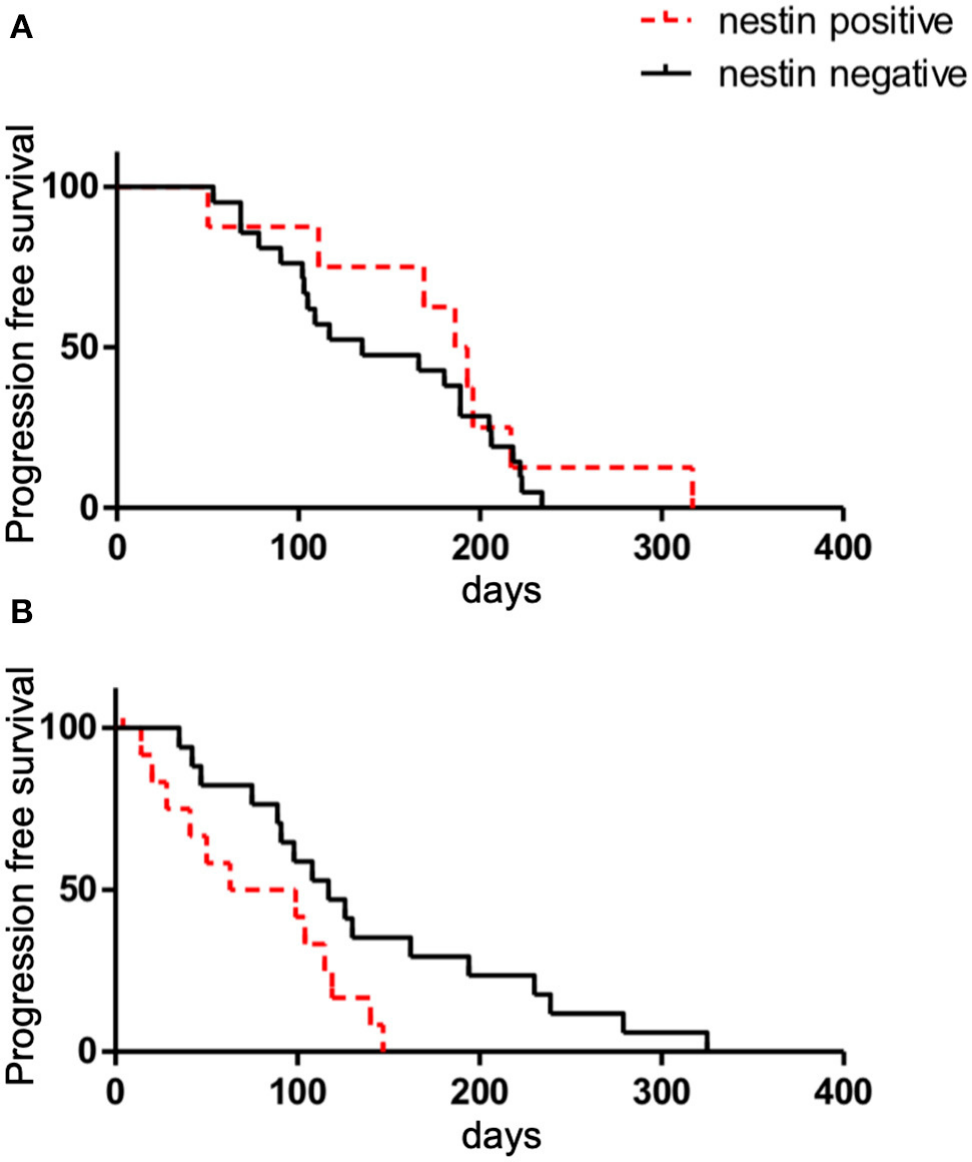
Kaplan-Meier survival curves of progression-free survival (PFS) according to nestin expression in patients with ED-SCLC treated with first-line chemotherapy **(A)** or all patients with recurrent disease treated with second-line chemotherapy **(B)**.

In terms of first-line chemotherapy for patients with ED-SCLC, no differences in RR or DCR were identified between patients with positive and negative nestin expression (RR: 87.5 vs. 85.0%, respectively; *P* = 1.00) (DCR: 87.5 vs. 85.7%, respectively; *P* = 1.00). Median PFS in patients with nestin-positive and -negative expression was 189.5 days [95% confidence interval (CI), 50–217 days] and 135 days (95%CI, 102–189 days), respectively (*P* = 0.402) (Figure 6A). In terms of second-line chemotherapy for patients with recurrent disease, the RR or DCR of patients with nestin-positive expression were also not significantly different from those of patients with nestin-negative expression (RR: 33.3 vs. 35.5%, *P* = 1.00; DCR: 58.3 vs. 76.5%, *P* = 0.42). Patients with nestin-positive expression experienced statistically shorter PFS (median PFS, 81 days; 95%CI, 20–119 days) compared to patients with nestin-negative expression (117 days; 95%CI, 75–194 days; *P* = 0.029) (Figure 6B). Best response could not be judged for two patients with ED-SCLC and nine patients with recurrent disease. PFS could not be judged for 1 ED-SCLC patient and eight patients with recurrent disease. Overall survival (OS) wasn't evaluated due to insufficient patient follow-up after 2nd line chemotherapy.

## Discussion

In this study, we showed that nestin promoted cell growth and reduced sensitivity to chemotherapy for SCLC *in vitro*, and was upregulated in chemotherapy-resistant cell lines. Furthermore, clinical expression of nestin was associated with the efficacy of chemotherapy in patients with recurrent SCLC. To the best of our knowledge, this is the first report to evaluate clinical expression of nestin, revealing a relationship with clinical outcome in SCLC patients.

Nestin is typically expressed in neuronal and myogenic precursors in the development stage (4). Recently, nestin has been detected in cancers arising from various lineages. Expression of nestin in cancer cells is reportedly associated with the malignant phenotypes, such as tumor growth, invasion and chemotherapy resistance. In addition, nestin was reported to be related to cancer stem cell phenotypes (14). We have previously reported that nestin is expressed in SCLC cells in association with neuroendocrine features and participates in malignant phenotypes, including cell growth and invasion (5). In this study, we confirmed the relationship between nestin expression and cell growth in both nestin-overexpressing cell lines *in vitro* and a mouse xenograft model. Moreover, we demonstrated nestin expression was related to chemoresistance and was upregulated in chemotherapy-resistant cell lines. These results suggest that nestin expression leads to chemotherapy resistance, especially acquired resistance in SCLC. This association between nestin and chemotherapy sensitivity has also been reported for hepatocellular carcinoma (6). One possible mechanism to account for the link between nestin expression and chemoresistance is epidermal-to-mesenchymal transition (EMT). Nestin is reportedly linked to chemoresistance through EMT in various cancers, including NSCLC (6, 15). On the other hand, the regulation of nestin is also considered to be important to acquired chemoresistance in SCLC. One recent report found that acquired chemoresistance in SCLC was controlled through an epigenetic regulator, the EZH2-SLFN11 axis, not through emergence of recurrent gene mutations or changes in copy number (16). Originally, differentiation of neurons and glial cells is regulated through EZH2 in the neural development phase (17). EZH2-overexpressing astrocytes revealed upregulation of the *Nestin* gene (18). EZH2 was also described to promote SCLC progression through suppression of the TGF-β-smad-ASCL1 pathway (19). We have previously suggested a relationship between ASCL1 and nestin (4). Inhibition of EZH2 led to down-regulation of nestin in glioblastoma (20). Considering these data, acquired chemoresistance in SCLC may be epigenetically led through EZH2-nestin signaling.

Immunohistochemical expression of nestin could be detected in SCLC tissues from clinical samples in this study. The rate of positive nestin expression in surgical specimens was higher than that in biopsy specimens. One reason could be that nestin expression was heterogeneous in cancer tissues (5, 21), resulting in a higher expression rate in surgical specimens that yield larger tissues compared to biopsy specimens. Immunohistochemical nestin expression was related to shorter PFS for second-line chemotherapy in this study. Associations between nestin expression and efficacy of chemotherapy have previously been reported for other cancer types (6, 8), and these results are in line with those findings. However, no significant difference in PFS following first-line chemotherapy were seen in this study. This might be due to the heterogeneity of nestin expression. Chemo-naïve SCLC is highly susceptible to chemotherapy, and most cells, including nestin-negative cells, would be killed by the first-line chemotherapy, leaving only nestin-positive chemotherapy-resistant cells to lead to relapse. Such a chemotherapy-induced selection of nestin-positive cells has been reported in a glioblastoma model (22, 23). In addition, nestin expression may be upregulated by exposure to first-line chemotherapy, considering the result in this study. Indeed, we encountered a case of an SCLC patient who showed nestin-negative tumor cells at initial diagnosis that became positive after first-line chemotherapy (not shown). Rebiopsy in more cases to examine nestin expression over time is needed to prove this hypothesis.

Several limitations need to be considered with the present study. First, this was a retrospective analysis of a small number of patients from a single institute. Therefore, we could not evaluate the differences of OS and confirm these results through multivariate analysis. Prospective investigation is needed to confirm the results of this study. Second, nestin expression could not be examined in biopsy specimens from 18 cases. Many SCLC specimens showed crush artifacts, and SCLC can be diagnosed from cytology in the clinical setting (24). Immunohistochemical evaluation of nestin expression can thus be difficult in routine clinical examination. Other modalities need to be considered to determine nestin expression clinically.

New therapeutic options for SCLC have been under development. For example, immune-checkpoint inhibitors (3, 25) and rovalpituzumab tesirine (26) have been investigated for SCLC. However, promising drugs remain few and far between, and the anticancer effects appear limited. The present results suggest nestin as a promising biomarker for predicting the effectiveness of chemotherapy or prognosis. Patients with nestin positive SCLC may need other treatment strategy than those with nestin negative SCLC. Nestin is a one of molecule markers of cancer stem cells. It was reported that cancer stem cells evade host immunesurveillance by PD-L1 expression (27). Therefore, anti-PD-1/PD-L1 therapy may be effective for nestin positive SCLC. In addition, nestin or nestin-related signaling may lead directly to a malignant phenotype and chemoresistance and may also provide new therapeutic targets in SCLC. The data from this study contribute to the understanding of the clinical function of nestin in SCLC. Further studies of nestin in SCLC are warranted.

## Data Availability Statement

The datasets generated for this study are available on request to the corresponding author.

## Ethics Statement

The studies involving human participants were reviewed and approved by the Institutional Ethics Committee of Nagoya City University Graduate School of Medical Sciences. The patients/participants provided their written informed consent to participate in this study. The animal study was reviewed and approved by Institutional Animal Care and Use Committee at Nagoya City University of Medical Science.

## Author Contributions

KS, KM, HI, and AN: conception and design. KS, EK, YuK, AT, and SF: cell line study. EK, YuK, and OT: animal study. AM and HI: immunohistochemistry study. KM, TU, SF, KF, YoK, HO, MT, YI, and TO: acquisition of clinical data. KS, KM, TO, and AN: interpretation of all data. All authors critically reviewed the manuscript and revised it. All authors approved the final version of the manuscript and agree to be accountable for all aspects of the work in ensuring that questions related to the accuracy or integrity of any part of the work are appropriately investigated and resolved.

## Conflict of Interest

The authors declare that the research was conducted in the absence of any commercial or financial relationships that could be construed as a potential conflict of interest.
